# Supplementary material for: Area efficient camouflaging technique for securing IC reverse engineering
Source: PLoS One. 2021 Nov 4;16(11):e0257679. doi: 10.1371/journal.pone.0257679 (PMC8568261; doi:10.1371/journal.pone.0257679)
Supplement: Attachment — Submitted filename: Response_to_Reviewers.pdf [file pone.0257679.s002.pdf]

A rebuttal letter

'Response to Reviewers

Editor Comments:

1. Please ensure that your manuscript meets PLOS ONE's style requirements, including those for file naming. The PLOS ONE style templates can be found at https://journals.plos.org/plosone/s/file?id=wjVg/PLOSOne_formatting_sample_main_body.pdf and https://journals.plos.org/plosone/s/file?id=ba62/PLOSOne_formatting_sample_title_authors_affiliations.pdf

Address: We have rechecked thoroughly the manuscript and corrected as per Plos template that is provided.

2. Please include captions for your Supporting Information files at the end of your manuscript, and update any in-text citations to match accordingly. Please see our Supporting Information guidelines for more information: http://journals.plos.org/plosone/s/supporting-information

Address: There is no supporting information. All data are within the manuscript.

3. If you did not receive any funding for this study, please state: “The authors received no specific funding for this work.”

Address: We have added in the cover letter.

4.Please complete your Competing Interests on the online submission form to state any Competing Interests. If you have no competing interests, please state "The authors have declared that no competing interests exist.", as detailed online in our guide for authors at http://journals.plos.org/plosone/s/submit-now.

Address: We have added in the cover letter (The authors have declared that no competing interests exist).

5. Please amend the manuscript submission data (via Edit Submission) to include authors Fakir Sharif Hossain and Md. Ismail Hossain.

Address: Thank you for the comment. We have added two more authors in the list.

6. Please review your reference list to ensure that it is complete and correct. If you have cited papers that have been retracted, please include the rationale for doing so in the manuscript text, or remove these references and replace them with relevant current references. Any changes to the reference list should be mentioned in the rebuttal letter that accompanies your revised manuscript. If you need to cite a retracted article, indicate the article’s retracted status in the References list and also include a citation and full reference for the retraction notice.

Address: Thank you for the comment. We have checked thoroughly and found ref. [12] should be replaced by ref. [40] in line 84.

Additional Editor Comments:

Address: Thank you so much for your valuable comments. We have added one more section named as "Related work and Motivation" in Line 42-116.

Reviewer comments

1) In line 30-32 the author mentioned "Another way of making camouflaged cells is to control the doping concentration in the drain area of a MOS gate so that the NMOS remains ON and the PMOS always remains OFF state, resulting in a NAND gate functioning as an inverter [12]"

>> Here the author mentioned an obfuscation process of disguising an inverter as a NAND gate using doping concentration. But ref [12] does not provide any such methods. The referencing seems wrong and must be updated.

Address: Thank you so much for the comments. We mistakenly did the wrong referencing. Sorry for the mistake. We have revised the reference and put the appropriate ref. of [40] in line 83-84. "**Another way of making camouflaged cells is termed as `covert gate' that leverages doping and dummy contacts to create camouflaged cells [40].**"

2) In line 90-92 From the designers or the defenders' point of view, some decisions are made, such as the number of camouflaged cells; the design affords what structure the camouflaged cells is to use in the design in what part of the design it may use these camouflaged cells.

>> This sentence contains grammatical error and need to be rewritten.

Address: Thank you so much for the comments. We have corrected the sentence in line 148-150 such as "**From the defenders' point of view, some decisions on camouflaged cells are made. The number of camouflaged cells the design affords with the structural usage and in what part of the design it may use these camouflaged cells."**

Reviewer #1: This paper explores IC obfuscation method by using true and dummy contact method. The authors have developed a open source obfuscated standard cell library that can be used to replace some of the standard cells representing critical logic to protect the IP from reverse engineering. Overall the idea was interesting but heavily depends on the fab.

Address: Thank you for the comment. We wholly agreed with you sir. In our future scope we may go for fab and also may try to improve the proposed library.

Reviewer #2: The study proposed a cost-effective camouflaging technique for two universal get NAND and NOR. It also proposed an open-sourced library with metal routing and dummy contact techniques.

The methodology, explanation, and experimental evaluation are well written. The theoretical explanation of how it takes minimal overhead is also satisfactory.
However, as the authors claim the proposed method outperforms the existing approaches with the experimental result, they should detail explain how fairly comparison takes place with different technology in Table-3 (even it compares in ratio or percentage of times) in terms of power, performance, and area? Also, in the comparison section, the explanation needs to explain more clearly from the reader's point of view. Like
1) how the values in line 329, 335, and 336 are drives from Table- 3?
2) more detail of the experimental setup for measuring the value of Table2,3 (detail of Table-1)
3)A traditional small-scale benchmark can also be considered for Table-3.

Address: Thank you for your comments. We have tried to address in our best.

1) For the more clarifications, we have rewritten the Comparison section in line number 379. We put some detail in this section according to TABLE-3 (line-381-391). Hopefully, the measuring value from Table-3 can be understandable.
2) We have added a detail explanation of tools used, experimental setup and how to calculate them in the result section from line 323 to 347.
3) We highly appreciate you direction on “A traditional small-scale benchmark can also be considered for Table-3”. We apologize, currently; we are unable to add them because it is out of scope right now. We do not have such data; however, we might consider it in our future scope.
