# Supplementary material for: Area efficient camouflaging technique for securing IC reverse engineering
Source: PLoS One. 2021 Nov 4;16(11):e0257679. doi: 10.1371/journal.pone.0257679 (PMC8568261; doi:10.1371/journal.pone.0257679)
Supplement: Attachment — Submitted filename: PLOS One Paper review.docx [file pone.0257679.s001.docx]

1) In line 30-32 the author mentioned “Another way of making camouflaged cells is to control the doping concentration in the drain area of a MOS gate so that the NMOS remains ON and the PMOS always remains OFF state, resulting in a NAND gate functioning as an inverter [12]”

>> Here the author mentioned an obfuscation process of disguising an inverter as a NAND gate using doping concentration. But ref [12] does not provide any such methods. The referencing seems wrong and must be updated.

2) In line 90-92 From the designers or the defenders’ point of view, some decisions are made, such as the number of camouflaged cells; the design affords what structure the camouflaged cells is to use in the design in what part of the design it may use these camouflaged cells.

>> This sentence contains grammatical error and need to be rewritten.
